# Behind closed doors. A case study exploring the lived experiences of a family of a person with the behavioral variant of frontotemporal dementia

**DOI:** 10.1177/14713012221126312

**Published:** 2022-09-27

**Authors:** Jeroen Bruinsma, Kirsten Peetoom, Frans Verhey, Christian Bakker, Marjolein de Vugt

**Affiliations:** Department of Psychiatry and Neuropsychology/Alzheimer Centre Limburg, School for Mental Health and Neuroscience, 5211 Maastricht University, The Netherlands; Department of Primary and Community Care, Radboud University Medical Centre, The Netherlands; Radboudumc Alzheimer Centre, The Netherlands; Groenhuysen, Centre for Specialized Geriatric Care, The Netherlands; Department of Psychiatry and Neuropsychology/Alzheimer Centre Limburg, School for Mental Health and Neuroscience, 5211 Maastricht University, The Netherlands

**Keywords:** frontotemporal dementia, caregiving, family, case study

## Abstract

**Objective:**

The behavioral variant of frontotemporal dementia is characterized by profound changes in personality and behavior that often start before the age of 65 years. These symptoms impact family life, particularly if (adult) children live at home. In research on young-onset dementia or frontotemporal dementia, the family itself is hardly ever a unit of analysis. Insight in the perspectives of different family members from the same household helps to obtain a deeper understanding of the complex impact of the symptoms on family dynamics.

**Methods:**

This case study explored the perspectives of one family having a relative with the behavioral variant of frontotemporal dementia living at home. Over the course of 4 months, different family members were individually interviewed twice. Two authors independently performed a directed content analysis.

**Results:**

The family consisted of a father, mother, and three adult children. Around 3 years before the interviews the father was diagnosed with frontotemporal dementia.

The main category identified was the change in family dynamics over the disease trajectory. Three subcategories characterized the changing family dynamics, namely (a) the change in existing roles, relationships and interaction patterns in the family due to early symptoms, (b) a redefinition of roles and responsibility in the family once the diagnosis was established, and (c) the formation of new roles, relationships and interaction patterns in the family by organizing post-diagnostic support at home.

**Conclusion:**

Symptoms of the behavioral variant of frontotemporal dementia have a complex and profound impact on family dynamics and change existing roles, relationships, and interaction patterns. Psychosocial support may help families by accounting for individual differences in involvement, coping, and bereavement. This may help to create a sense of mutual understanding between family members that could potentially strengthen their relationship. This may help families to deal with the difficult challenge of organizing care for a relative with frontotemporal dementia who lives at home.

## Introduction

The behavioral variant of frontotemporal dementia is characterized by changes in personality and behavior, such as apathy, loss of empathy, uninhibited behavior, compulsiveness, lacking social insight, and absence of disease awareness (Ducharme et al., 2020; Rabinovici & Miller, 2010). Although the symptoms can start at older age, the majority of the persons with frontotemporal dementia have a symptom onset before the age of 65 years. Therefore, frontotemporal dementia is considered to be a specific subvariant of young-onset dementia (Nunnemann et al., 2012; Van de Veen et al., 2021). The impact of the behavioral symptoms with family life is profound. Especially at young age, when persons with the behavioral variant of frontotemporal dementia and their spouses are likely to be employed and may have children living at home (Caceres et al., 2016). Typically, the early symptoms start insidious and are often misattributed for work-related stress, psychiatric symptoms, marital issues, or as the result of a midlife crisis (Ducharme et al., 2020; Bruinsma, Peetoom, Bakker et al., 2020). This complicates a timely diagnosis and results in high levels of pre-diagnostic uncertainty in family members (Van Vliet et al., 2011). Obtaining a diagnosis can help family members to understand the changes in their relative with the behavioral variant of frontotemporal dementia (Bruinsma, Peetoom, Bakker et al., 2020; Tookey et al., 2021). Additionally, for family members the diagnosis often confirms the irreversibility of symptoms (de Vugt & Verhey, 2013). This can lead to feelings of grief in anticipation of losing their relative to the symptoms (Tookey et al., 2021).

For family members, disclosure of a the diagnosis often marks the beginning of identifying themselves as caregivers (Bruinsma, Peetoom, Bakker et al., 2020; de Vugt & Verhey, 2013). At a young age, it is often a spouse who becomes the primary caregiver (Tookey et al., 2021). This can cause shifts in the distribution of responsibility and changes in interpersonal relationships (Caceres et al., 2016; Kaizik et al., 2017). To illustrate, persons with the behavioral variant of frontotemporal dementia tend to show only little affection towards others. In turn, spouses may experience a diminished sense of couplehood (Bruinsma, Peetoom, Millenaar et al., 2020; Holdsworth & McCabe, 2018). For (adult) children, the child-parent relationship may reverse as children gradually become more responsible for their parent with frontotemporal dementia (Kaizik et al., 2017; Oyebode et al., 2013). Children in puberty, adolescence or (young) adulthood may worry about the burden on their healthy parent and may postpone plans such as moving out or studying because of their involvement in care (Cartwright et al., 2021; Millenaar et al., 2014). Although caring for a parent with young-onset dementia can be stressful, it can also have a positive effect on children who may feel to mature faster as they adopt more responsibility. Children can also experience stronger bonds with other relatives (Grundberg et al., 2021).

Although specific symptoms of the behavioral variant of frontotemporal dementia can impact family life, there is only little understanding of the experiences of children living at home (Caceres et al., 2016; Tookey et al., 2021). In most research, the family itself is hardly ever a unit of analysis. Exploring the perspectives of different family members from the same household (i.e., children and parents) allows a detailed insight in the dynamic context in which individual experiences emerge when organizing care together (Chapman et al., 2019; Kumamoto et al., 2004). More specifically, investigating individual experiences of different family members helps to deeper understand changes in roles, relationships, and interaction patterns (Jokogbola et al., 2018; Kindell et al., 2014). This may help to identify directions to support the family as a whole (Kilty et al., 2019; Roach et al., 2013). Currently, most support does not account for family dynamics and is often designed with a primary caregiver, Alzheimer’s dementia, or old age in mind (Kobiske & Bekhet, 2018; Mayrhofer et al., 2018; Tookey et al., 2021). Therefore, this study explores the lived experiences of one family who has a relative with the behavioral variant of frontotemporal dementia who lives at home.

## Methods

A case study design was used to examine the perspectives of different members from the same family. This allowed an in-dept understanding of the complex interaction between the symptoms and the consequences of the behavioral variant of frontotemporal dementia for family dynamics. Between April and August 2021, two rounds of individual interviews were conducted with individual family members. Follow-up interviews after 4 months enabled an insight in their experiences over time. The consolidated criteria for reporting qualitative research (COREQ) were used to report the findings (Tong et al., 2007).

### Recruitment

Inclusion criteria were (a) the person with the behavioral variant of frontotemporal dementia was younger than 65 years at symptom onset and was living at home, (b) the spouse and at least one adult child were willing to participate, (c) at least one of the children was living at home, and (d) the diagnosis was established at least 6 months ago to be able to reflect on the adaptation process following the diagnosis. Additionally, a selection criterion was that the person with the behavioral variant of frontotemporal dementia was eligible for participation to account for the patient perspective (Gove et al., 2018; Griffin et al., 2016).

Recruitment took place via healthcare organizations affiliated with the Dutch Young-onset Dementia Knowledge Center (Kenniscentrum Dementie op Jonge Leeftijd) and the Dutch frontotemporal dementia peer-support organization (FTD lotgenoten). Healthcare professionals and caregivers linked to these organizations were asked if they were involved with a family of a person with the behavioral variant of frontotemporal dementia, and to inquire if the family was willing to participate. One family was recruited, and the first author had a telephone call with the potential participating family to inform them about the study. Before inclusion, all participating family members gave written consent for participation.

### Data collection

Individual interviews were preferred over group interviews to ensure that family members were able to voice their own perspective (Reczek, 2014). The initial semi-structured interviews lasted around 60 min and were conducted in separate rooms at the parental house to ensure privacy. To safeguard that participants could tell their own story it was possible for them to exclude sensitive information from the analysis.

To guide the initial interviews, a topic list was developed based on data from earlier conducted focus group discussions (Bruinsma, Peetoom, Bakker et al., 2020) and interviews with relatives of persons with the behavioral variant of frontotemporal dementia conducted in the Needs in Young onset Dementia (NeedYD) study (Van Vliet et al., 2010). The topic list was discussed with caregivers of persons with frontotemporal dementia who were not included in this study to substantiate the interview questions. The final topic list asked family members to reflect on different phases of the disease trajectory, namely the pre-diagnostic phase, the diagnostic trajectory, the present, and the future. Questions aimed to openly explore perceptions, emotions, and experiences regarding role division, relationships, interaction patterns, personal goals, expectations, and the distribution of responsibility.

Four months after the initial interviews, follow-up interviews were conducted to deepen the findings by exploring the lived experiences of the family over time and clarify ambiguities by member checking (Carter et al., 2014). A 4-month follow-up was specified to limit the impact on the participating family members. The follow-up interviews lasted around 60 min. The children not living at the parental house were willing to engage in the follow-up interviews but given time restraints they preferred to be interviewed via a video call, using Zoom or Microsoft Teams.

### Data analysis

Interviews were audio-recorded and transcribed verbatim. Fieldnotes were made about interactions in-between the interview sessions. After the initial interviews, the first and second authors independently performed a directed content analysis by means of open coding in Atlas.ti (Hsieh & Shannon, 2005; Savin-Baden & Major, 2013). In a directed content analysis theoretical concepts are used to collect data and direct the coding process (Hsieh & Shannon, 2005). Therefore, the topic list and coding process were structured according to phases of the disease trajectory (i.e., the pre-diagnostic phase, the diagnostic trajectory, the present, and the future) (Bruinsma, Peetoom, Bakker et al., 2020). The first and second author organized discussion sessions to reach consensus on the used codes (Bradley et al., 2007). Codes and (sub)categories following the initial interviews were visualized in a mind-map to support discussion among the first, second, and last authors to summarize the main findings (Suter, 2012). Then, findings were discussed in several meetings with the entire research team to substantiate the results and formulate directions for the follow-up interviews. In the follow-up interviews, participants reflected on the past 4 months. Subsequently, the main findings were briefly discussed, and questions were asked to deepen the findings and clear ambiguities. Again, the first and second authors independently coded the transcripts from the follow-up interviews and discussed how this substantiated the findings.

### Trustworthiness

By interviewing family members twice, this study allowed for a more comprehensive picture of their experiences. This increased the credibility of findings as it allowed to ask additional questions, clear ambiguities, and shortly discuss results to deepen our findings (Suter, 2012). This allowed for member checking of the main findings and preliminary conclusions (Merriam, 2009; Tracy, 2010). To account for researcher bias, all analyses were performed independently by two researchers and the results were discussed extensively with the entire research team to establish investigator triangulation (Carter et al., 2014). More specifically, in monthly meetings the research team reflected on the process of the data collection, the analysis, findings, and conclusions. Members of the research team had varied backgrounds including nursing, health sciences, (neuro)psychology, and neuropsychiatry. All researchers were experienced in conducting qualitative research with caregivers of persons with young-onset dementia and the behavioral variant of frontotemporal dementia.

### Ethical considerations

The study protocol was approved by the Research Ethics Committee of the Faculty of Health, Medicine and Life Sciences of Maastricht University, the Netherlands (FHML-REC/2020/102).

## Results

### Case description

One family was eligible for participation. The family lived in a rural area of the Netherlands, and consisted of a father, mother, and three children. The father of the family was diagnosed with the behavioral variant of frontotemporal dementia around 3 years before the initial interviews. At the time of the interviews, the family was supported by a dementia casemanager and the father attended daycare for 4 days a week. The mother of the family was employed parttime. The children (two sons, and one daughter) were aged in their late-20s and early-30s. The oldest son lived at his parents’ house and took over the family business after the diagnosis. His siblings had left the parental house around 10 years before the diagnosis. The father having the behavioral variant of frontotemporal dementia was unable to participate in our study given the intensity of symptoms and lack of disease insight.

#### Early symptoms

The family expressed that, in retrospect, the exact onset of the first symptoms was difficult to pinpoint but likely started around 8 years before the diagnosis of the behavioral variant of frontotemporal dementia was established. This was attributed to the insidious start, slow progression, and subtle changes in the character and behavior of their father. Looking back on the pre-diagnostic phase, the family noticed symptoms such as apathy, lack of empathy, hoarding, uninhibited eating, and changes in social behavior, which are characteristic for the behavioral variant of frontotemporal dementia.

#### Current symptoms

The behavioral symptoms of frontotemporal dementia had intensified in the years following the diagnosis. At time of the first interviews, the family coped with daily apathetic, stereotypic, and uninhibited behavior of the father. To illustrate, the father watched TV for hours, was fixated on food and eating, repeatedly sang one-liners, hoarded items, and performed rituals by repeatedly closing doors and turning off the lights. According to the family the key symptoms were the lack of disease insight and loss of emotional responsiveness. To illustrate, the father denied that he was ill, had difficulty to recognize changes in his behavior, and was unable to understand the beliefs, intentions, and emotions of others.

During the follow-up interviews after 4 months, the family noticed that impairment in cognitive functioning had worsened. For instance, they mentioned disorientation in time, inability to understand basic everyday facts, and lacking ability to process information.

### Changing family dynamics over the disease trajectory

The directed content analysis resulted in one main category, namely a change in family dynamics over the disease trajectory. Three subcategories were identified that characterize the change in family dynamics, namely (a) the change in existing roles, relationships, and interaction patterns in the family due to early symptoms, (b) a redefinition of roles and responsibility in the family once the diagnosis was established, and (c) the formation of new roles, relationships, and interaction patterns in the family by organizing post-diagnostic support at home.

#### Change in existing roles, relationships, and interaction patterns in the family due to early symptoms

Pre-morbid characteristics of the father influenced coping with early symptoms. Before symptoms of frontotemporal dementia had onset, there was a certain level of stability in roles, responsibility, and interaction patterns. The father was the ‘pater familias’. He was the head of the family and ran the family business. The family lived up to his expectations and standards. When early symptoms of frontotemporal dementia had insidiously started, his directive leadership made it difficult to ignore his strong opinion, and to question his behavior. This led to friction between the father and his daughter who had left the parental house on a young age. Later on, his role became more indifferent, indecisive, and less involved. The family noticed these changes but struggled to take over responsibility, given his authority. This led to uncertainty in the mother and adult children. To illustrate, the oldest son experienced an internal struggle regarding his position and his future within the family business.

“My father always knew what he was doing but gradually I got doubts like ‘Does it add up to what he does?’, ‘Why am I still here?’, and ‘Should I leave?’. However, before I was able to leave, things had to be arranged but that was his responsibility. So basically, I was stuck.”

**– Oldest son –**

The apathetic and indifferent behavior of the father adversely influenced the relationships with his family members. Relationships with him became remote and emotionally distanced due to his lack of social responsiveness. To illustrate, he showed no empathy and was hardly interested in others. In turn, the mother experienced that the relationship with her husband lacked reciprocity. This made her feel neglected and she thought about filing for divorce.

“My husband never treated me badly, but he was authoritarian and laid down the law. Slowly this changed and he started to agree more. This was very unsatisfying because I had the impression he simply was not interested in me anymore.”

**– Mother –**

The apathetic behavior of the father changed the interaction patterns within the family as communication with him became superficial. Also, he denied and trivialized the apathetic behavior. In turn, the other family members increasingly started to discuss their concerns with each other. This was accelerated when they noticed changes in the social behavior of the father. More specifically, he became more selfish and started to make road trips without being able to explain his destination.

Based on the conversations about the father’s behavior, the family came to the joint decision to consult the general practitioner (GP). The father denied symptoms and refused to go to the GP, but the family was very concerned about his behavior and his driving abilities. Therefore, the oldest son took a leadership role and took his father’s car keys as leverage to visit the GP.

“I was shaking, wondering how he was going to react after I took his car keys. His reaction was ‘Just give me the keys back. Just give them back’. Non-stop. This continued for days. I stood my grounds to force him to visit the general practitioner.”

**– Oldest son –**

Upon consultation, the GP noticed social inappropriate behavior of the father in the waiting room and immediately referred to a specialized neurologist. The diagnosis of the behavioral variant of frontotemporal dementia followed upon neuropsychological assessment and an MRI-scan.

#### A redefinition of roles and responsibility in the family once the diagnosis was established

The diagnosis facilitated a better understanding of symptoms and clarified the apathetic behavior of the father having frontotemporal dementia. This allowed for a new perspective on relationships, roles, and responsibility.

The mother realized the deterioration in the quality of their relationship was not her husband’s fault. The diagnosis made her aware that her role had changed into being the primary caregiver. This insight strengthened her in taking the lead in the decision-making process about care options. This was an important insight because her husband had always been the one making the important decisions in the family.

“The diagnosis is devastating because there is no cure and symptoms progress. However, the diagnosis is also a relief. […] I thought ‘now it’s me who is going to make the decisions.’ More as caregiver than as his wife.”

**– Mother –**

The diagnosis also helped the daughter to reevaluate the relationship with her father. She reflected on the love-hate relationship they previously had. This led to the insight she had distanced herself from her relatives in the last years, and now had a more passive role within the decision-making process.

“The positive effect of the diagnosis was that it helped me realize our complicated relationship was not only my fault.”

**– Daughter –**

After disclosure of the diagnosis the family reevaluated their expectations towards the father. This allowed the children to define new roles and take responsibility. For example, the sons felt that they had to take on a more supportive role to help their mother in organizing care. The oldest son took the lead in the family business and felt strengthened to make decisions without consulting his father.

“The diagnosis made me realize it was not my father who could make the decisions anymore. We were in charge. He kept saying ‘No, no, no. You don’t have to do this’. However, to get things done you have to ignore him. In this sense, taking his car keys was already a good practice.”

**– Oldest son –**

#### Formation of new roles, relationships, and interaction patterns in the family by organizing support

In the years after the diagnosis, the dynamics in the family further changed due to the fact that all care and support was organized at home. Although in the decision-making process regarding care the mother was primarily responsible, the sons were closely involved. This led to role reversal as the mother and children now shared authority over daily decisions, instead of the father. This had improved the communication and strengthened the relationship among the family members directly involved in organizing care. On the other hand, the relationship to the father had drastically changed by the progressing behavioral symptoms of frontotemporal dementia.

“My father basically is part of the furniture. It is difficult to stay emotionally attached to him. We feed him, supervise him, and he pulls of a happy façade.”

**– Oldest son –**

Organizing support together had created a sense of cohesion between the family members directly involved. The youngest son also helped his older brother with running the family business. This had strengthened the sense of companionship between the brothers.

“In the phase following the diagnosis everything changed for the positive. We started to talk about what we were going to do with the family business. If you talk about it, you go for it together. This gave us energy.”

**– Middle son –**

The daughter felt more distant from organizing care. She studied and experienced it as confronting to see her father deteriorate. According to the daughter, she applied an emotional coping style and needed more space to grieve than her brothers. Therefore, she deliberately distanced herself from the care situation at home. Her mother had noticed this and tried to protect her daughter by not burdening her with discussing care related matters.

“I think everyone has different needs in coping with this. […] I do not necessarily need to cope with this the same way my brothers do. I need more space to grieve.”

**– Daughter –**

On the contrary, the mother and sons were able to emotionally detach from the situation. Their high level of involvement was grounded in using similar coping strategies. They were very creative in adapting their daily lives and they formed an alliance in organizing care at home for as long as possible, even though they were uncertain about the future. This created cohesion between them.

“We already placed a lock on the refrigerator. If necessary, we will store the knifes behind a lock too. We would even get rid of the stove if he would turn on the gas. We are very adaptive. He [my father] taught us to be resourceful himself.”

**– Oldest son –**

## Discussion

### Key results

This case study allowed a unique insight in the changing family dynamics that accompany having a relative with the behavioral variant of frontotemporal dementia. The findings reveal that early symptoms slowly altered the role and position of the father within his family. His apathetic behavior and low levels of emotional responsiveness adversely affected the relationships with his family. Obtaining the diagnosis allowed the family to understand the apathetic behavior and the emotionally distanced relationship with their father having the behavioral variant of frontotemporal dementia. This helped the family to reevaluate their expectations, allowed to redistribute responsibility, and redefine new roles. The mother and sons formed a strong alliance and were practical in organizing the daily care. This created cohesion, improved their communication, and strengthened the relationships between them. In contrast, the daughter was more distanced from organizing care. She had more need to cope emotionally and needed space for bereavement.

### Interpretation of findings

Similar to prior research, in our study insufficient role fulfilment of the person having the behavioral variant of frontotemporal dementia led to pre-diagnostic role ambiguity in family members (Kilty et al., 2019). We found that early symptoms affected the role, level of involvement, and the position of the person having frontotemporal dementia within the family system. More specifically, family members experienced ambiguity about the cause of the apathetic behavior of their relative with undiagnosed frontotemporal dementia. Subsequently, this led to ambiguity about their own role, responsibility, and the future. For example, the oldest son experienced that the family business deteriorated due to his father’s apathic behavior but he struggled to take responsibility because it was difficult to transcend his father’s authority. This pre-diagnostic role ambiguity is probably directly related to the characteristic symptoms of the behavioral variant of frontotemporal dementia, such as apathy and impaired social cognition (Ducharme et al., 2020; Russell et al., 2020). Similar to our findings, these symptoms affect relationships of persons with the behavioral variant of frontotemporal dementia as interactions become more remote and emotionally distanced (Holdsworth & McCabe, 2018; Tookey et al., 2021). Without the diagnosis these symptoms of apathy and emotional bluntness are easily mistaken for selfishness (Massimo et al., 2013). In turn, family members may blame their relative with the behavioral variant of frontotemporal dementia for early symptoms, which can adversely affect the experienced level of closeness (Holdsworth & McCabe, 2018; Paton et al., 2004).

In our study the family had postponed visiting the GP. It is known that early symptoms of the behavioral variant of frontotemporal dementia are particularly difficult to label as signs of a pathology (Bruinsma, Peetoom, Bakker et al., 2020; Rasmussen et al., 2019). Postponing help-seeking behavior in the pre-diagnostic phase can contribute to the diagnostic delay (Van Vliet et al., 2011, 2013). Typically, the early symptoms of the behavioral variant of frontotemporal dementia are also difficult to recognize for GPs. This is attributed to the young age and the symptomatic overlap with psychiatric disorders (Beber & Chaves, 2013; Giamarelou et al., 2020). Our findings show the diagnosis was accelerated because the GP referred to a specialized neurologist after noticing impairments in social cognition such as social awkward behavior in the waiting room. Confirming previous research findings, obtaining the diagnosis was important for the family as it facilitated understanding and acceptation of symptoms (Bruinsma, Peetoom, Bakker et al., 2020; de Vugt & Verhey, 2013). Prior work has documented that obtaining the diagnosis may also introduce new uncertainty, for example regarding the future (Grundberg et al., 2021; Tookey et al., 2021). In contrast, we found the diagnosis positively helped the family to plan for the future. More specifically, the diagnosis helped the family with reevaluating expectations, redistribute responsibility, and redefine new role patterns. This empowered the family to transcend the strong opinion and authority of their relative having the behavioral variant of frontotemporal dementia. This helped them to take responsibility in the decision-making process. Until now, only limited research has reported these positive aspects that accompany diagnostic disclosure (Tookey et al., 2021).

Although (adult) children may not perceive themselves as caregivers, they are often an essential part of the support structure for persons with young-onset dementia or the behavioral variant of frontotemporal dementia living at home (Cartwright et al., 2021; Grundberg et al., 2021). We found that organizing care together increased cohesion, strengthened interaction and communication, and created a sense of companionship between family members directly involved. The adult children in our study experience a reversed child-parent role and similar as documented in other studies they adopted increasingly more responsibility (Hall & Sikes, 2018; Kaizik et al., 2017). Although this can be stressful and may have a negative impact on the future development of children (Johannessen et al., 2015; Millenaar et al., 2014), adopting more responsibility can also have positive effects as children may grow into a leadership role and perceive to mature (Cartwright et al., 2021). Coping strategies can influence how children cope with the changing role patterns, intensifying symptoms, and feelings of bereavement (Grundberg et al., 2021; Millenaar et al., 2014). Feelings of grief were particularly linked to symptoms that had changed the personality and behavior of the father with frontotemporal dementia. To cope with grief, some children may become increasingly involved whereas others may distance themselves from the caregiving situation as a mechanism of self-protection. Both are normal mechanisms to cope with bereavement and are frequently used by children who have a parent with young-onset dementia (Cartwright et al., 2021). Our findings show that the level of engagement in organizing caregiving was related to the position of the children in the pre-diagnostic phase, the use of coping strategies, and having the ability to emotionally detach from the person having frontotemporal dementia. To illustrate, in the pre-diagnostic phase the daughter was more distanced from the situation at home, compared to the oldest son who lived at home and worked in his father’s business. After the diagnosis, he was already closely involved and therefore able to take over responsibility and form an alliance with his mother and brother in organizing care. In contrast, after the diagnosis the daughter needed more space to emotionally cope with her feelings of bereavement and deliberately distanced herself from the care situation. In turn, she was less involved in the decision-making process. A direction for the future is to support families by accounting for these individual differences in involvement, coping, and bereavement caused by the dynamic interplay between symptoms and family life. Creating a sense of mutual understanding between family members could potentially strengthen the relationships and help families to deal with the difficult challenge of organizing care for a relative with the behavioral variant of frontotemporal dementia who lives at home.

### Strengths and limitations

The case study design allowed a unique insight in the complex interplay between the symptoms and consequences of the behavioral variant of frontotemporal dementia and changes in family dynamics. The case study design helped to get a detailed understanding of the individual perspectives of different family members involved. For studying such complex phenomena a case study can be particularly useful (Flyvbjerg, 2006). Our study aimed to bridge a knowledge gap by using the family as a unit of analysis and adhere to a holistic and family-centered approach (Roach et al., 2013). It was not our aim to provide generalizable claims about the impact of the behavioral variant of frontotemporal dementia on family life and we recognize the complexity and uniqueness of each family, person, and care situation.

Interviewing the family twice over the course of 4 months allowed an iterative process between data collection and analysis. This enabled member checking the findings and allowed for a unique insight in the experiences of caregivers over time. A study with an even longer interval would allow a more detailed understanding of the experience of caregivers, for example by exploring the transition towards institutionalization. A limitation of this study is it did not include the perspective of the person with the behavioral variant of frontotemporal dementia. Adhering to recommendations of Alzheimer Europe the person with dementia was opted to participate in our study (Gove et al., 2018). However, in our case the participation was complicated by the intensity of symptoms. Previous research has demonstrated that persons with the behavioral variant of frontotemporal dementia also experience profound changes in interpersonal relationships and interaction patterns with their relatives (Griffin et al., 2016). Therefore, including a family in the phase directly after the diagnosis may allow for the inclusion of persons with frontotemporal dementia in research. This remains a direction for future research.

## Conclusion

Our findings confirm that symptoms of the behavioral variant of frontotemporal dementia have a profound impact on family life. Particularly, behavioral symptoms and deficits in social cognition changed existing roles, relationships, and interaction dynamics. This started already in the years before the diagnosis. Complicating is that the diagnosis of the behavioral variant of frontotemporal dementia is often delayed, resulting in a long pre-diagnostic phase. In this phase, relationships diminish as the role and position of the person with frontotemporal dementia within the family is gradually changing. Disclosure of the diagnosis allowed for a sudden redefinition of roles and helped to redistribute responsibility within the family. Post-diagnostic support should focus on supporting caregivers to cope with these abrupt shifts. A system approach could focus on supporting all family members, for example by accounting for the individual differences in positions, involvement, coping, and need for bereavement. This may help to create a sense of mutual understanding between family members and could potentially strengthen the relationships and improve organizing care together. Our findings showed that organizing care together had a range of positive effects for family members such as creating cohesion, improving communication, and strengthening relationships.
